# Continuous predictive mortality risk monitoring after allogeneic hematopoietic stem cell transplantation

**DOI:** 10.1038/s41598-026-57782-x

**Published:** 2026-06-18

**Authors:** Nick Rucks, Sergej Korlakov, Sebastian Alexander Scharf, Anna Rommerskirchen, Rainer Haas, Stefan Conrad

**Affiliations:** 1 Department of Computer Science, Faculty of Mathematics and Natural Sciences, Heinrich Heine University Düsseldorf, Universitätsstraße 1, 40225 Düsseldorf, Germany; 2 Institute of Medical Microbiology and Hospital Hygiene, Medical Faculty and University Hospital, Heinrich Heine University Düsseldorf, Universitätsstraße 1, 40225 Düsseldorf, Germany; 3 Department of Hematology, Oncology, and Clinical Immunology, Medical Faculty and University Hospital Düsseldorf, Heinrich Heine University Düsseldorf, Moorenstraße 5, 40225 Düsseldorf, Germany; 4 Solventum Germany GmbH, Edisonstraße 6, 59174 Kamen, Germany

**Keywords:** Allogeneic stem cell transplantation, Artificial intelligence, (Remote) Patient monitoring, Clinical decision support systems, Prognostic and health management, Explainable AI (XAI), AI in oncology, Cancer, Computational biology and bioinformatics, Medical research, Oncology

## Abstract

Allogeneic hematopoietic stem cell transplantation remains critical for treating high-risk hematological malignancies like leukemia. Despite advances in treatment, early mortality remains clinically significant, with approximately 6–11% of patients dying within the first 100 days after transplantation. This highlights the need for dynamic monitoring strategies beyond static pre-transplant risk assessments. This study introduces a novel, interpretable, real-time proof-of-concept monitoring framework that continuously assesses individualized mortality risk during treatment using routinely collected clinical data. The framework employs deep learning models to predict seven-day mortality risk based on 22 laboratory parameters from the previous 14 days and five demographic features. The framework incorporates an explainability method that provides time-resolved insights into predictions, which can be aggregated across time intervals or patient groups for broader interpretation. We evaluated the approach on data from 891 patients treated at the University Hospital of Düsseldorf (UKD; 2004–2019), as well as on an independent cohort derived from the MIMIC-IV database. Our experiments demonstrate that the predicted mortality risk aligns with observed outcomes, achieving a patient-level AUROC of 0.95 in the primary (UKD) cohort. Preliminary expert evaluation suggests that the predictions and explanations are intuitive and clinically relevant, supporting awareness of complications and highlighting potential for timely intervention.

## Introduction

Allogeneic hematopoietic stem cell transplantation (HSCT) is a treatment for patients with high-risk hematological malignancies including certain subtypes of acute leukemia^1^. The principle of HSCT is to eradicate malignant cells in the recipient and restore hematopoiesis using stem cells from a healthy donor. In Europe, 19, 806 HSCTs from 694 centers were reported in 2021, with a steady upward trend interrupted only during the SARS-CoV-2 pandemic^2^.

Prior to transplantation, patients undergo conditioning therapy consisting of chemotherapy with or without radiotherapy^3^, followed by infusion of donor stem cells to reconstitute hematopoiesis. During the early post-transplant period, patients are highly vulnerable to severe complications such as infections, graft-versus-host disease (GvHD), and relapse ^4^. These complications can evolve rapidly and are reflected in continuously changing clinical parameters, requiring close monitoring and timely clinical intervention.

**Fig. 1:**
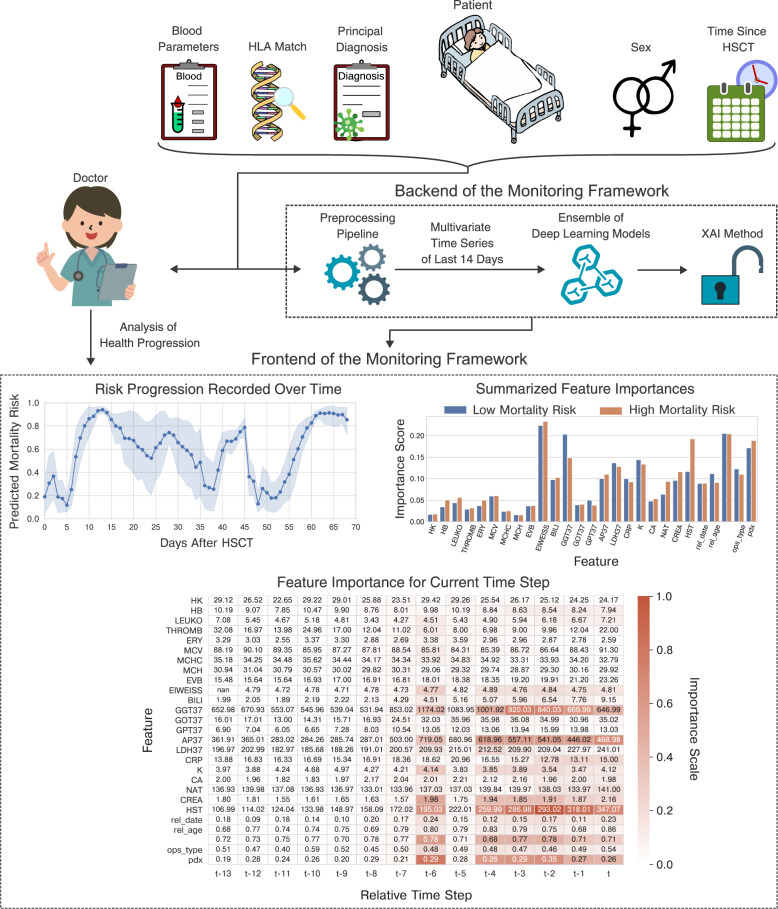
Schematic representation of the proof-of-concept monitoring framework. It is designed to continuously monitor patients’ health within the first 100 days after HSCT by analyzing routinely collected clinical data from the last 14 days. These data are preprocessed into an equidistant multivariate time series and fed into an ensemble of deep learning models. The models are used to estimate a mortality risk score between zero and one, representing the probability of death within the next seven days. In addition, an explainability module is applied to the model outputs to assign an importance score to each feature at every time step over the last 14 days. The framework is intended to present the estimated risk score and feature importances per time step, as well as summarized feature importances over multiple time steps in a frontend, with the aim of providing data-driven insights to support medical professionals during treatment. The figure was created by the authors using Inkscape and public-domain graphical elements; no AI-generated content was used.

Despite continuous advancements in treatment, early mortality remains clinically significant, with approximately 6–11% of patients dying within the first 100 days following HSCT^5^. The mortality occurring within this time frame is categorized as early mortality and is often attributable to treatment-related complications or relapse^6^. Consequently, a number of approaches have been proposed for the prediction of mortality following HSCT in general or in specific conditions^7–11^. However, these approaches rely solely on pre-transplant data to make an estimate of overall post-transplant mortality, having a limited utility beyond day zero, i.e., the day of cell infusion. Therefore, physicians currently have no option but to rely on their own clinical experience to assess patients’ evolving mortality risk at this stage. An explainable system capable of dynamically assessing day-to-day mortality risk after day zero would provide valuable support in clinical decision-making. By enabling continuous surveillance, such a system could enhance physician’s awareness of emerging complications, allowing timely preventive interventions and more efficient allocation of healthcare resources.

Nevertheless, developing such a system presents significant challenges due to the lack of open-source datasets. Hence, researchers must collect sufficient amount of data from hospitals for the training of machine learning models and conduct an extensive data cleaning process, which requires close supervision by experts.

Aside from these challenges, existing patient health monitoring approaches for alternative use cases, that explicitly utilize temporal information from time series data, have shown promising results. For instance, the method developed by Jacobsen et al.^12^ uses data from clinical wearables to detect clinical complications in patients undergoing treatment for hematologic malignancies. Another approach proposed by Nitski et al.^13^ enables monitoring the long-term probability of mortality due to post-transplantation complications in liver transplant recipients. Beyond providing a risk score for specific complications, the authors also incorporated explainability techniques to analyze the most important features.

In the context of HSCT, a recent study by Spohr et al.^14^ introduced a dynamic risk stratification method that uses a separate random forest model for each of the first 30 days after HSCT to classify whether a patient will die within 100 days, presenting results in discrete risk categories. While this approach provides long-term outcome predictions, it ultimately informs physicians only about the risk of death within a broad 100-day horizon. In contrast, we present a proof-of-concept framework for real-time monitoring with a short predictive window, estimating the risk of death within the next seven days based on routinely collected laboratory parameters from the preceding 14 days. This shift in focus from long-term outcome classification to short-term risk monitoring aims to provide additional actionable information for daily clinical decision-making and requires different model architectures, preprocessing strategies, and explanation techniques.

The proposed monitoring framework is illustrated in Fig. 1. The main contributions of this work to the area of health informatics research are:

- A proof-of-concept framework for continuous, explainable assessment of individualized all-cause mortality risk within 100 days after HSCT based on routinely collected clinical data.
- A demonstration of the feasibility of dynamic, time-resolved mortality risk monitoring using multivariate clinical time series and deep learning models, along with an exploration of its potential for risk stratification.
- A detailed description of the implementation process, including data preprocessing, model selection and the choice of explainability method, with a focus on potential applicability in clinical practice.

## Materials and methods

Developing a reliable framework for monitoring mortality risk in post-transplant patients requires methods that capture the complexity of patient risk trajectories. These trajectories can be dynamically influenced by the treatment and vary between patients. We address this challenge in two key ways. First, we treat the input data as multivariate time series, preserving temporal dependencies across multiple clinical variables. Second, we formulate the prediction task as a rolling short-term risk estimation problem: predicting mortality within the next seven days based on data from the preceding 14 days. This formulation reflects a clinically relevant monitoring scenario, where risk must be continuously reassessed as new data becomes available. Instead of reducing predictions to binary outcomes, we retain the model’s continuous output (a score between zero and one), allowing for a more nuanced and time-resolved assessment of mortality risk.

Feature selection was guided by the availability and consistency of routinely collected clinical data across patients, with a focus on parameters commonly measured during post-transplant care. This ensures that the proposed framework relies on features that are broadly available in clinical practice and reduces the risk of bias arising from feature-specific missingness.

All methods and experiments were performed in accordance with the relevant guidelines and regulations.

### Data

The primary clinical cohort was collected at the University Hospital Düsseldorf (UKD) and used for model development, including training, validation, and internal evaluation. The external cohort, derived from the MIMIC-IV database^15^, was used exclusively for external testing to assess the generalizability of the proposed framework and was not involved in model training or parameter tuning.

In both cohorts, we included only adult patients ($\ge 18$ years) who underwent allogeneic HSCT. No patients were prospectively enrolled, and for the UKD cohort, no interventions outside of standard clinical care were performed.

#### UKD cohort

The UKD cohort comprises clinical routine data (electronic health records, EHR) from 891 adult patients who underwent at least one allogeneic HSCT at the University Hospital of Düsseldorf between 2004 and 2019. It was compiled from two data sources to include both patient demographic data and diagnoses alongside laboratory values. As a result, the data contain both numerical time series and categorical variables. Patients who underwent autologous HSCT were not included.

All patients were hospitalized during transplantation and the early post-transplant phase as part of standard clinical care, but not necessarily throughout the full 100-day monitoring period. Some patients were readmitted during this period. After discharge, laboratory values were recorded during regular outpatient follow-up visits and, when applicable, during readmission. Accordingly, the frequency and density of available laboratory measurements vary across patients depending on hospitalization status, follow-up schedule, and individual clinical course.

The data were pseudonymized in line with the HIPAA Safe Harbor requirements. Specifically, all 18 categories of direct identifiers were removed, and patients are identified by double-blind assigned integer identifiers not related to their data. In addition, time-related information is presented only relative to the date of HSCT, and demographic characteristics such as age are grouped into sufficiently broad categories to prevent re-identification of individuals, including those with unusual characteristics or outlier status. The data made available for this manuscript therefore contain no information that could be used to identify participants.

The use of the UKD cohort was approved by the Ethics Committee of the Medical Faculty at Heinrich Heine University Düsseldorf (case-id: 2019-513) on August 12, 2019. The approval permits processing of the de-identified data used in this study. In light of this approval, the removal of all identifiers according to HIPAA Safe Harbor, and the secondary nature of the data use, we believe that no individual written informed consent is required for this study.

#### MIMIC-IV cohort

To evaluate the robustness and generalizability of the proposed framework, we additionally used an independent cohort extracted from the publicly available MIMIC-IV database^15^, which contains de-identified health-related data from intensive care unit (ICU) patients. Access to the database requires completion of a data use agreement and certification process. From this database, we identified patients who underwent HSCT based on procedure and diagnosis codes and constructed a cohort designed to mirror the UKD setting as closely as possible. Mappings of laboratory parameters to MIMIC-IV itemids and ICD procedure codes used for cohort identification are provided in Supplementary Tables S1 and S2.

However, notable differences in data availability required adaptations in feature extraction. In contrast to the more standardized data collection in the HSCT cohort, measurements in MIMIC-IV are recorded based on clinical necessity in an intensive care setting. As a result, several laboratory parameters that are routinely monitored after HSCT, including gamma-glutamyl transferase (GGT37), C-reactive protein (CRP), and total protein (EIWEISS), exhibit substantial missingness, with many patients lacking these measurements entirely.

This missingness is not random but reflects clinical decision-making, where laboratory tests are performed selectively depending on the patient’s condition. Consequently, the absence of these parameters may itself carry implicit clinical information, but their inconsistent availability limits their direct use in a standardized modeling pipeline.

In addition to laboratory features, transplant-specific categorical variables are also affected. In particular, donor and graft information (ops_type), which encodes HLA matching and donor relationship in the primary cohort, is not available in MIMIC-IV and cannot be reliably reconstructed from the available data.

To maintain consistency of the input representation while avoiding bias from feature availability, we adapted the preprocessing accordingly. For the laboratory parameters GGT37, CRP, and total protein, values were imputed using the mean value of the respective feature computed over the first 100 days post-HSCT, independent of the number of available measurements per patient. This approach allows the incorporation of these features without disproportionately favoring patients with more frequent laboratory testing. For ops_type, we assigned a fixed worst-case category corresponding to an unrelated donor without HLA matching for all patients in the MIMIC-IV cohort.

Overall, these differences highlight the challenges of transferring models between datasets with distinct clinical contexts and documentation practices.

### Data preprocessing

Clinical routine data after HSCT are characterized by irregular sampling, variable feature availability, and treatment-dependent measurement frequency. These challenges are further amplified when combining data from different clinical settings, such as the UKD and MIMIC-IV cohorts described above. Therefore, a carefully designed preprocessing pipeline is required to ensure comparability across patients and over time.

**Fig. 2:**
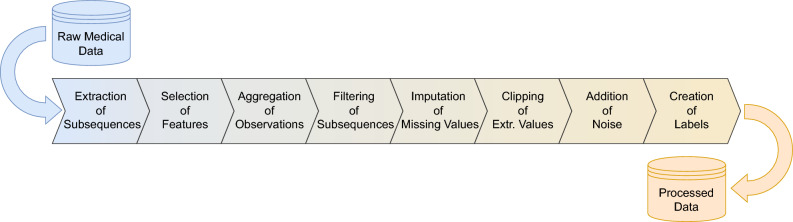
Schematic visualization of the preprocessing pipeline. It shows each processing step applied to the raw data to obtain the data used for model training.

The preprocessing includes data from the time interval of $-14$ to 107 days relative to the transplantation day zero in order to predict the daily mortality risk within the interval of 0 to 100 days. We did not apply normalization or standardization to the data, as all patients share the same initial conditions on day zero, and we aim to preserve the natural interactions between features. The preprocessing pipeline consists of eight steps, as illustrated in Fig. 2. The following sections provide a detailed explanation of each step.

#### Extraction of subsequences

To create the required input for training and evaluation of our monitoring framework, we first excluded data of the day of death for the deceased patients. We then applied a sliding window of 14 days length with a step size of one day, moving it across the time series to extract subsequences for each patient. The first iteration of the sliding window starts at day $-13$, whereas the final iteration extends to day 100.

Consequently, predictions made on day zero for the next seven days are based on data from the time interval $[-13,0]$ days. In contrast, predictions on day 100 for the interval [101, 107] rely on data from days 87 to 100. This sliding window formulation enables continuous reassessment of patient risk and reflects a real-time monitoring scenario in which predictions are updated as new data become available.

The window size of 14 days was chosen for three reasons. First, it ensures that the conditioning phase prior to transplantation is fully captured. Second, since engraftment typically begins within 10–14 days after HSCT, this window spans both the transplantation and early engraftment phases. Third, given that clinical follow-ups typically occur at least once per week, a 14-day window provides sufficient observations to capture temporal trends.

#### Selection of features

Blood-related features play an important role in tracking and assessing patients’ risk trajectories post-HSCT. Since different complications require the analysis of specific laboratory parameters, the recorded features vary among patients. As a result, some parameters are documented for only a small subset of individuals, whereas others are consistently recorded for all patients. Furthermore, the admission status of patients introduces additional variability in the type, amount, and frequency of recorded parameters.

To prevent the deep learning models from learning outcome-related biases due to feature availability, we prioritize features that are consistently available across patients in the HSCT-specific UKD cohort. Based on these considerations, we selected 22 laboratory parameters that are routinely measured and broadly available during the first 100 days after HSCT. These variables capture key aspects of organ function, inflammation, and hematopoietic recovery and are commonly used in post-transplant monitoring.

While most selected laboratory parameters are consistently recorded in the UKD cohort, some variables exhibit substantial missingness in the external MIMIC-IV cohort, where measurements are recorded based on clinical necessity in an intensive care setting. In particular, gamma-glutamyl transferase (GGT37), C-reactive protein (CRP), and total protein (EIWEISS) are not available for all patients. To retain these clinically relevant features while ensuring compatibility across cohorts, we incorporate them as described in “Data” section.

Additionally, we considered five categorical features, namely the relative day of prediction, age, sex, principal diagnosis, and donor/graft information. The relative day provides temporal context. Age and sex were included due to known differences in immune response and baseline laboratory values^16^. Since both cohorts contain only adult patients, age was grouped into three categories (18–29, 30–60, and $>60$ years) to avoid overfitting to specific values^17^ while preserving clinically meaningful stratification. Principal diagnoses were grouped into malignant, non-malignant, and other conditions. A comprehensive overview of the primary diagnoses and their respective frequencies is presented in Supplementary Table S3. Donor/graft information was summarized into four categories based on HLA matching and donor relationship.

In the MIMIC-IV cohort, donor and graft information (ops_type) is mostly missing and cannot be reliably reconstructed. To maintain a consistent feature space, it is therefore approximated as described in Section Data.

While additional clinical variables such as graft-versus-host disease (GvHD), conditioning intensity, infection status, or vital signs are known to influence post-transplant outcomes, these variables were not consistently available in structured form across cohorts. Including them would have introduced bias and reduced the number of usable patient risk trajectories. Therefore, this work prioritizes robustness and reproducibility over maximal feature richness, focusing on consistently recorded or reliably approximated variables. Incorporating richer clinical variables represents an important direction for future work.

All selected features and categorizations were approved by medical experts at the University Hospital of Düsseldorf and are listed in Table 1. Descriptive statistics of the features after preprocessing are presented in Table 2 and Table 3.

**Table 1:** Abbreviated feature names and their full descriptions used for the monitoring framework.

Abbr.	Feature Name	Abbr.	Feature Name
AP37	Alkaline phosphatase at 37 $^{\circ }$C	BILI	Bilirubin
CA	Calcium	CREA	Creatinine
CRP	C-Reactive protein	EIWEISS	Total protein
ERY	Erythrocytes	EVB	Erythrocyte volume distribution
GGT37	Gamma-glutamyl transferase at 37 $^{\circ }$C	GOT37	Glutamic-oxaloacetic transaminase at 37 $^{\circ }$C
GPT37	Glutamate pyruvate transaminase at 37 $^{\circ }$C	HB	Hemoglobin
HK	Hematocrit	HST	Urea
K	Potassium	LDH37	Lactate dehydrogenase at 37 $^{\circ }$C
LEUKO	Leukocytes	MCHC	Mean corpuscular hemoglobin concentration
MCV	Mean corpuscular volume	NAT	Sodium
THROMB	Thrombocytes/Platelets		
rel_date	Relative date post-HSCT	rel_age	Patient age (grouped)
sex	Patient sex	ops_type	Donor and graft information (grouped)
pdx	Principal diagnosis, underlying pathology (grouped)

**Table 2:** Descriptive statistics for the UKD and MIMIC-IV cohorts across the selected laboratory features and patient age. For each feature, the table reports the number of recorded measurements and the mean with standard deviation.

Dataset	UKD		MIMIC-IV	
Feature name	Number of measurements	Mean ± SD	Number of measurements	Mean ± SD
Alkaline phosphatase at 37$^{\circ }$C	35,281	110.87 ± 93.30	7,031	99.04 ± 70.46
Bilirubin	45,170	1.21 ± 2.34	7,058	0.95 ± 1.48
Calcium	42,830	2.21 ± 0.17	7,952	2.12 ± 0.14
Creatinine	49,289	0.93 ± 0.43	8,104	0.95 ± 0.68
C-Reactive protein	48,543	3.48 ± 5.61	14	6.96 ± 6.81
Total protein	29,368	6.04 ± 0.88	542	5.43 ± 0.71
Erythrocytes	52,201	3.21 ± 0.48	7,936	2.83 ± 0.42
Erythrocyte volume distribution	51,512	16.57 ± 2.96	7,893	17.10 ± 3.33
Gamma-glutamyl transferase at 37$^{\circ }$C	40,747	153.95 ± 247.67	35	133.26 ± 138.93
Glutamic-oxaloacetic transaminase at 37$^{\circ }$C	41,012	35.91 ± 98.29	7,048	38.85 ± 297.15
Glutamate pyruvate transaminase at 37$^{\circ }$C	40,927	44.72 ± 80.29	7,049	42.17 ± 110.32
Hemoglobin	52,216	9.67 ± 1.59	7,959	8.82 ± 1.25
Hematocrit	53,405	28.44 ± 4.92	8,160	25.55 ± 3.79
Urea	46,565	42.75 ± 30.08	8,076	22.83 ± 20.71
Potassium	51,334	4.19 ± 0.51	8,242	4.04 ± 0.48
Lactate dehydrogenase at 37$^{\circ }$C	41,482	279.21 ± 263.94	6,488	295.59 ± 461.16
Leukocytes	52,199	3.84 ± 4.77	7,700	3.51 ± 4.55
Mean corpuscular hemoglobin	52,199	30.17 ± 2.14	7,936	31.30 ± 2.31
Mean corpuscular hemoglobin concentration	52,198	34.10 ± 1.28	7,937	34.57 ± 1.66
Mean corpuscular volume	52,198	88.61 ± 7.18	7,936	90.77 ± 7.61
Sodium	49,676	138.09 ± 3.91	8,220	137.74 ± 3.64
Thrombocytes/Platelets	52,089	72.97 ± 84.27	9,359	70.58 ± 73.09
Patient age	891	51.21 ± 12.24	182	53.60 ± 12.28

**Table 3:** Distribution of categorical variables in the UKD and MIMIC-IV cohorts, together with category-specific mortality within 100 days after HSCT. For each variable and category, the table reports the number and percentage of patients, as well as the number and percentage who died within 100 days.

Dataset	Category	UKD		MIMIC-IV	
Variable		Patients, n (%)	100-day deaths, n (%)	Patients, n (%)	100-day deaths, n (%)
Sex	Male	519 (58.2)	49 (9.4)	107 (58.8)	4 (3.7)
	Female	372 (41.8)	44 (11.8)	75 (41.2)	6 (8.0)
Underlying pathology	malignant diseases	685 (76.9)	75 (10.9)	120 (65.9)	5 (4.2)
	non-malignant diseases	198 (22.2)	15 (7.6)	47 (25.8)	3 (6.4)
	other diseases	8 (0.9)	3 (37.5)	15 (8.2)	2 (13.3)
Donor graft information	HLA-identical, unrelated donor	509 (57.1)	55 (10.8)	0 (0.0)	0 (0.0)
	HLA-identical, related donor	219 (24.6)	12 (5.5)	0 (0.0)	0 (0.0)
	Non-HLA-identical, unrelated donor	137 (15.4)	17 (12.4)	182 (100.0)	10 (5.5)
	Non-HLA-identical, related donor	26 (2.9)	9 (34.6)	0 (0.0)	0 (0.0)
Age groups	younger than 30 years	75 (8.4)	7 (9.3)	11 (6.0)	0 (0.0)
	30–60 years	589 (66.1)	65 (11.0)	104 (57.1)	5 (4.8)
	older than 60 years	227 (25.5)	21 (9.3)	67 (36.8)	5 (7.5)
Mortality 100 days post-HSCT	survived	798 (89.6)	–	172 (94.5)	–
	died	93 (10.4)	–	10 (5.5)	–

#### Aggregation of values

Some of the selected 22 laboratory parameters were measured more than once for the same patient on the same day. In such cases, we used the median value to obtain a single daily measurement per feature, reducing the influence of extreme values and measurement noise. We note that the frequency of measurements may itself carry clinical information. However, incorporating this explicitly would introduce treatment-related bias, as patients in poorer condition are typically monitored more frequently as discussed in “Adding noise” section.

#### Filtering of Subsequences

Given the importance of temporal trends for prediction, we excluded subsequences with fewer than two observations for a laboratory parameter within the 14-day window, as they do not allow reliable estimation of temporal dynamics. This threshold represents a trade-off between retaining sufficient data and ensuring meaningful temporal information within each subsequence.

For the MIMIC-IV cohort, the same criterion was applied at the subsequence level. However, due to substantial missingness of certain laboratory parameters (GGT37, CRP, and total protein), these features were imputed with cohort-level mean values as described in “MIMIC-IV cohort” section. Therefore, these variables primarily contribute contextual information rather than temporal dynamics, while temporal patterns are captured by the remaining features. As a result, we obtained approximately 88, 500 unique subsequences for the UKD cohort and 7, 208 for the MIMIC-IV cohort.

#### Imputation

The numerical features within each subsequence are irregular and vary in length. To obtain a uniform representation suitable for model input, missing values were imputed using a combination of last observation carried forward, next observation carried backward, and linear interpolation, depending on the position of missing values within the sequence. This approach preserves temporal continuity while minimizing the introduction of artificial trends.

#### Clipping

We observed that some patients exhibited extreme laboratory values. To prevent such outliers from disproportionately influencing model training while preserving their clinical relevance, we applied percentile-based clipping. Specifically, for each feature, values below the $0.5\text {th}$ percentile and above the $99.5\text {th}$ percentile were clipped to these respective thresholds, computed separately for each cohort (UKD and MIMIC-IV). This preserves the relative ordering of values while limiting the impact of extreme outliers.

#### Adding noise

Both cohorts exhibit treatment-related bias reflected in measurement frequency: patients in poorer condition are typically monitored more frequently than stable patients. Although aggregation and imputation reduce this effect, residual patterns may still be present. To mitigate this bias, we added Gaussian noise (mean 0, standard deviation 0.05) to each measurement, constrained within feature-specific value ranges. This encourages a deep learning model to focus on temporal patterns in the data rather than implicitly encoding measurement frequency.

#### Creation of labels

Each subsequence was labeled as *deceased* if the patient died within seven days following the last day of the subsequence, and as *survived* otherwise. For example, a subsequence covering days $[-13, 0]$ is labeled as deceased if death occurs within days [1, 7]. Similarly, a subsequence from the time interval of day 87 to day 100 is labeled as *deceased* if the patient died within the interval from day 101 to day 107.

This labeling strategy aligns with the objective of short-term risk prediction in a clinical monitoring setting, enabling early detection of patient deterioration while aiming for a clinically actionable lead time. The resulting dataset is highly imbalanced, with approximately $1\%$ of subsequences labeled as *deceased*.

### Prediction models

During the selection process of an appropriate deep learning model for our framework, we identified the following key requirements. The chosen model must capture temporal dependencies in multivariate time series data, ensuring, it could learn and recognize patterns across time from clinical laboratory parameters. Additionally, the model has to generalize well on the limited dataset of 891 patients and at the same time scale effectively when the dataset grows. Another consideration is the computational efficiency, i.e., our model needs to be compact enough to run locally, without requiring extensive computational resources. Based on these requirements, we identified the Explainable Convolutional Neural Network for Multivariate Time Series Classification (XCM)^18^ as the optimal choice.

In addition to the XCM, we considered a Long Short-Term Memory Network (LSTM)^19^ and a Multilayer Perceptron (MLP)^20^. In contrast to CNNs, which were originally developed for image classification, the LSTM architecture was explicitly designed for time series data and is commonly used as a baseline for time series classification tasks in the medical domain^21–25^. The MLP architecture, initially conceived to handle non-linearly separable data, serves as another baseline for comparing performance of XCM with an approach that is not specifically designed or adapted to handle multivariate time series data. Similar to the LSTM, it is also a widely employed model for classification tasks in the medical domain^26–30^.

### Explainability method

The explainability method aims to enable physicians to understand the reasoning behind the predictions without requiring technical expertise. The explanation method should be capable of providing explanations fast enough to avoid interfering with everyday medical practice. Further, the calculated explanations should allow a quick visual identification of the relevant parameters. In addition, the explanation method should use the original model and being applicable to original data. These requirements guided us to the application of Integrated Gradients (IG)^31^ in combination with Temporal Saliency Rescaling (TSR)^32^ in order to increase the interpretability of the explanations.

IG is a gradient-based explainability method that attributes feature importance by integrating gradients along a path from the baseline input to the actual input. Since IG was originally developed for explaining image classification tasks, it does not account for temporal dependencies between data points. To address this limitation, we applied TSR to the output of IG. TSR computes a time relevance score and a feature relevance score, whose product yields the time-resolved feature importance score.

### Cross-validation and ensemble of models

In our study, we employed a 10-fold cross-validation strategy with an 8-1-1 split, ensuring that within each iteration, $80\%$ of the patients were used for training, $10\%$ for validation, and $10\%$ for testing. Given the limited size of the dataset of 891 patients, where around $10\%$ of patients died within the first 100 days, the split was chosen to maximize the training data available whereas ensuring a robust evaluation of the model performance. To further assess the generalization capabilities of our models, we repeated this cross-validation procedure five times, each time using new random assignment of patients to folds without replacement, while ensuring that the label distribution remained consistent across all folds. Since the predictions of individual models vary depending on the training data, we aimed to provide physicians with uncertainty estimates for each mortality risk prediction to further support their decision-making. To compute uncertainty estimates for a given patient *p* at time step *t*, we selected the model from each cross-validation run where *p* was in the test fold, i.e., where the patient was not used for training. This procedure resulted in five models per patient and time step, to yield five independent mortality risk predictions that we used to compute the mean risk score and the corresponding $95\%$ confidence interval. Since the analysis of five explanations with the size of $27 \times 14$ each can be time-consuming and impractical, we average and normalize the five explainability results for each patient *p* at time step *t* to provide a unified explanation while preserving the findings.

For our experiments, all models were trained for 25 epochs using the preprocessed data described above and a weighted loss function that mitigates the massive label imbalance. Predicted risk scores were further calibrated by temperature scaling on the validation fold of the respective run ^33^.

## Experiments and results

The primary objective of this study was to evaluate whether continuous mortality risk predictions derived from routinely collected clinical data could support the monitoring of patients during the first 100 days post-HSCT. To this end, we assessed both the predictive performance of the proposed models and their potential utility for identifying patients at increased risk of short-term mortality. We analyzed (i) how accurately the models estimated seven-day mortality risk, (ii) whether these predictions could be aggregated into meaningful patient-specific risk progression curves for outcome stratification, and (iii) whether rising risk may provide support for early detection and clinical intervention. In addition, we assessed the clinical plausibility of the model explanations through an expert reviewed case study including twenty patients and further evaluated performance on an external MIMIC-IV cohort.

**Fig. 3:**
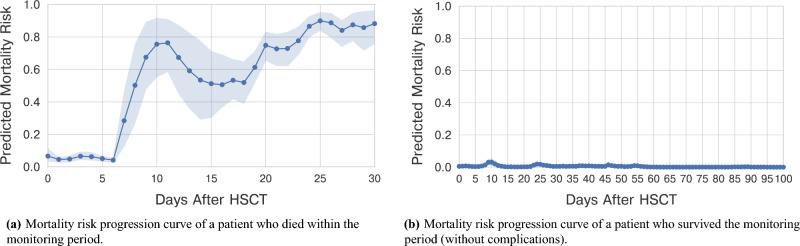
Example mortality risk progression curves for patients who (**a**) died and (**b**) survived during the monitoring period. The blue line shows the mean predicted mortality risk, and the light blue area indicates the $95\%$ confidence interval.

The framework produces daily updated mortality risk scores that can be aggregated into patient-specific risk progression curves, enabling a longitudinal view of all-cause mortality risk post-HSCT. Representative examples of such trajectories for patients who died and for patients who survived are illustrated in Fig. 3.

### General patterns of predicted mortality risk

**Fig. 4:**
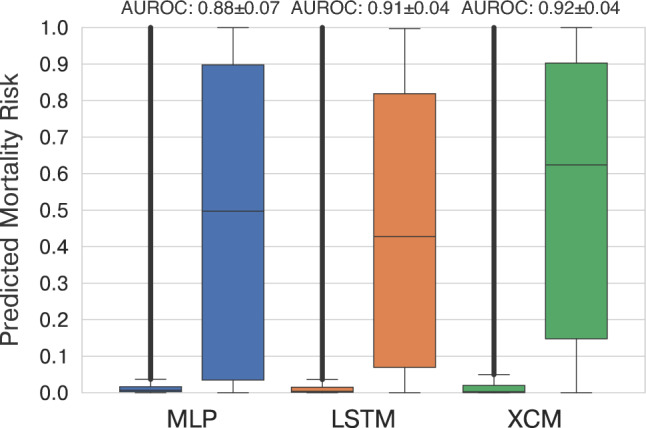
Distributions of predicted mortality risks for the next seven days labeled as survived (left) and died (right) for each model. Discrimination is quantified by mean and standard deviation. AUROC is computed across 50 folds.

All models assigned higher mortality risk scores to subsequences preceding death within the seven day prediction window than to all other periods, as shown in Fig. 4. The figure illustrates the distributions of predicted mortality risks for subsequences in which the patient died within seven days (right) and for all other subsequences (left) for each model type.

Among the evaluated model types, XCMs exhibited the strongest separation between high-risk and low-risk subsequences. They achieved the highest median predicted risk ($63\%$) for subsequences preceding death and showed the largest interquartile separation between outcome groups. Quantitatively, the XCMs outperformed both baselines in predicting seven day mortality within the first 100 days post-HSCT, achieving an AUROC of $92\% \pm 4\%$, a sensitivity of $56\% \pm 22\%$, and a specificity of $97\% \pm 2\%$. These results indicate that the predicted probabilities were well aligned with observed mortality outcomes and captured meaningful differences between high-risk and low-risk states.

**Fig. 5:**
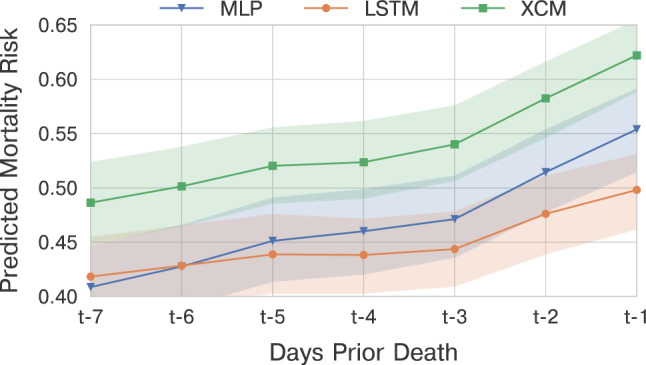
Temporal progression of predicted mortality risk, averaged across all patients who died, during the seven days preceding death. Day *t* denotes the day of death.

Additionally, we observed that the predicted mortality risk from all models increased within the seven days preceding the patient’s death, as demonstrated in Fig. 5. The predicted risk was consistently higher when death was imminent than at earlier time points and showed a continuous upward trend in the days leading up to death. Although the models were trained only for binary seven-day mortality prediction, they appeared to capture temporal patterns beyond simple characteristics in laboratory parameters that anticipated mortality risk.

These dynamics explain the distinct risk trajectories observed in Fig. 3. Patients who died exhibited higher and more volatile risk scores, whereas survivors maintained consistently low risk levels with smaller variance and stronger agreement between models, reflected in narrower confidence intervals.

**Fig. 6:**
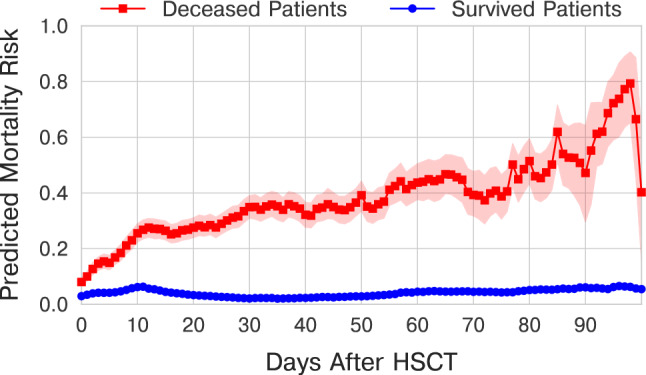
Average predicted mortality risk progression curve for patients who died (red) and survived (blue).

To assess these patterns at the population level, we averaged the risk progression curves across all patients who died and survived, as shown in Fig. 6. The resulting curves illustrate a clear separation between the two groups, with patients who died consistently exhibiting higher average predicted risk than survivors from day zero onward. This separation becomes more pronounced over time, particularly beyond the early post-transplant phase. During the first 21 days after HSCT, patients who died showed higher model importance for urea (HST), creatinine (CREA), and liver related parameters (BILI, GGT37, GPT37, GOT37), whereas survivors showed higher importance for C-reactive protein (CRP) and general blood count parameters (HB, LEUKO, THROMB, ERY). Because predictions at day zero were based on the preceding 14 days, these differences may partly reflect variation in the conditioning, however, this could not be assessed directly because conditioning related variables were not available in the data. Additional discussion regarding feature importance is provided in “Explainability and clinical interpretation of model predictions” section.

### Patient-level risk stratification

Previous analyses showed that predicted mortality risk scores were higher in patients who died and increased as death approached. We therefore assessed, whether predicted seven-day mortality risk could stratify patients at the individual level and identify high-risk cases during the first 100 days after HSCT. If these risk estimates are correlated with actual critical patient status, patients who eventually died should have shown an elevated risk score at some point before death. We therefore examined in how many patients an alarm would have been triggered by the system, indicating that a patient could have been identified as being at increased mortality risk.

For this analysis, we formulated a patient-level prediction task in which patients were classified as high-risk if their predicted mortality risk exceeded a threshold of 0.5 at least once during the first 100 days post-HSCT, and as low-risk otherwise. This threshold reflects a clinically interpretable cutoff, corresponding to a predicted probability of death within the next seven days exceeding $50\%$.

This task was best solved by the XCM models, which achieved strong predictive performance at the patient level, with an AUROC of $95\%$, a sensitivity of $73\%$, and a specificity of $92\%$, as showen in Table 4. Moreover, the XCM models yielded a false alert rate of approximately 1:1, indicating that for each correctly identified high-risk patient, only one false alert (false positive) was generated. Analysis of the 63 false positive alerts revealed that a substantial proportion of these cases actually corresponded to clinically relevant situations. Specifically, $51\%$ of patients identified as false positives were admitted to the intensive care unit during the corresponding stay, and $22\%$ died during the same hospital stay but beyond the 100-day observation window. These findings suggest that many false positive alerts may reflect true clinical deterioration that was not captured by the evaluation definition, rather than purely incorrect predictions.

In contrast, the models failed to identify 25 patients as high-risk who died (false negatives). However, in 17 of these patients, the predicted mortality risk increased before death but did not exceed the predefined threshold of 0.5. This suggests that the models captured worsening risk in many of these cases, but the fixed threshold was too conservative to trigger an alert. These findings highlight the sensitivity of the results to the chosen cutoff and suggest that alternative alerting strategies may improve detection in future work.

**Table 4:** Predictive performance of the models for patient-level outcome prediction within 100 days post-HSCT, reported as mean ± standard deviation over $n=50$ folds.

Model	Precision	Recall	F1	Specificity	AUROC	AUPRC
MLP	$0.42 \pm 0.13$ (0.48)	$0.70 \pm 0.22$ (0.66)	$0.51 \pm 0.14$ (0.56)	$\mathbf {0.88 \pm 0.05}$ (0.92)	$0.90 \pm 0.06$ (0.91)	$0.69 \pm 0.13$ (0.66)
LSTM	$0.39 \pm 0.14$ (**0.53**)	$0.68 \pm 0.22$ (0.67)	$0.48 \pm 0.13$ (0.59)	$0.87 \pm 0.07$ (**0.93**)	$0.92 \pm 0.05$ (0.94)	$0.68 \pm 0.15$ (0.71)
XCM	$\mathbf {0.44 \pm 0.14}$ (0.52)	$\mathbf {0.76 \pm 0.20}$ (**0.73**)	$\mathbf {0.53 \pm 0.11}$ (**0.61**)	$0.87 \pm 0.07$ (0.92)	$\mathbf {0.93 \pm 0.04}$ (**0.95**)	$\mathbf {0.73 \pm 0.11}$ (**0.74**)

Bold indicates best results. Values in parentheses represent performance when predictions are averaged across models, as used for generating risk progression curves.

#### Sensitivity analysis using maximum risk scores

We further investigated detection performance by considering the maximum predicted mortality risk for all patients across the five predictions obtained through cross-validation. This reflected a setting in which the treating physician aimed to minimize the risk of missing a high-risk patient while accepting a higher number of false alerts. Using this strategy, sensitivity increased substantially for all model ensembles (XCM, LSTM, and MLP), with each exceeding $90\%$. For the XCM models, only six patients who died were missed, whereas the LSTM and MLP models each missed nine patients. This improvement in sensitivity came at the cost of an increased false alert rate of approximately 3 : 1. The models did not consistently miss the same patients, e.g., a single patient (id: 448) was not identified as high-risk by any model, with a maximum predicted mortality risk of $18\%$. As discussed in the expert evaluation (“Assessment of model predictions and explainability by an expert physician” section), this case illustrates a limitation of the approach when deterioration is not clearly reflected in the recorded laboratory values.

### Exploring potential for timely clinical intervention by early mortality risk detection

While the previous analysis showed that the proposed framework could stratify patients according to their overall mortality risk, its practical relevance also depends on whether high-risk patients could be identified early enough to support clinical intervention.

To assess this, we analyzed the timing of high-risk alerts relative to their death date. Among correctly identified patients, $66\%$ had a lead time of more than seven days before death, whereas only $13\%$ were identified less than three days before death. These findings showed that, despite the short seven-day prediction horizon, the framework often identified high-risk patients with meaningful lead time before death.

**Fig. 7:**
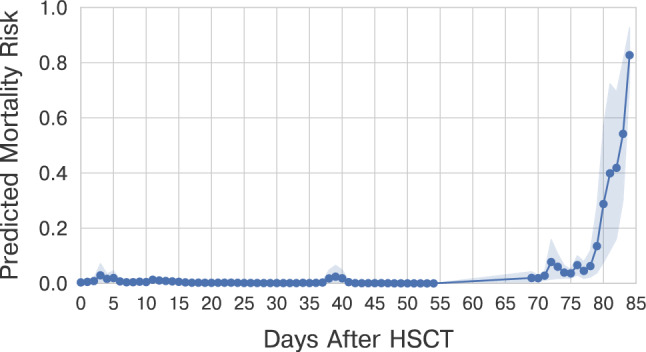
Example of increasing predicted mortality risk in a patient who was not transferred to the ICU prior to death.

A related question was whether such alerts merely reflect already apparent clinical deterioration, for example in patients who were escalated to intensive care. To explore this, we examined patients who died without a recorded ICU stay during the corresponding hospitalization. Among these 14 patients ($15\%$), five were identified as high-risk by the framework before their death. In an additional four cases, the predicted mortality risk showed a consistent upward trend before death but did not exceed the alert threshold. These findings suggest that the framework can detect worsening risk in patients without documented ICU escalation and may therefore provide an useful additional escalation signal for physicians during patient monitoring. Figure 7 shows an example in which the predicted mortality risk increased significantly before death in a patient who was hospitalized but not admitted to the ICU.

### Explainability and clinical interpretation of model predictions

To facilitate clinical interpretation of the predicted mortality risks, we applied Integrated Gradients in combination with Temporal Saliency Rescaling to compute feature importance scores for each prediction (see “Explainability method” section for details). Since each patient and time step were associated with five XCM predictions from the cross-validation experiment, we averaged and normalized the corresponding explanation scores to obtain a single explanation for each time step.

**Fig. 8:**
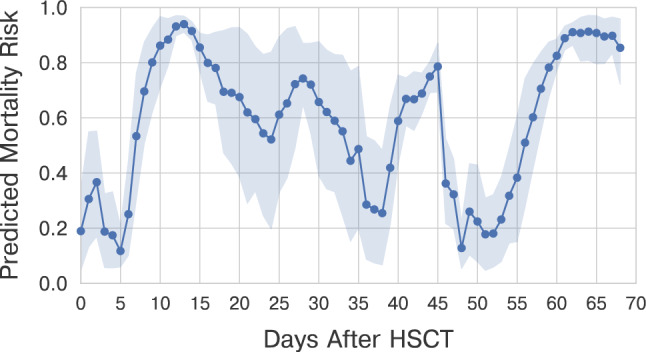
Mortality risk curve for patient *P* (id: 664), showing two distinct phases of increasing predicted mortality risk during the observed period.

To illustrate how these explanations can support clinical reasoning, we analyzed a representative case (patient *P*, id: 664), who exhibited multiple pronounced increases in predicted mortality risk and for whom complete longitudinal data were available. The corresponding risk progression curve is shown in Fig. 8. The patient remained hospitalized throughout the observation period and died on day 69 post-HSCT. Two distinct phases of increasing mortality risk were observed: an early increase between days 5 and 13, and a later, more pronounced increase between days 52 and 68.

**Fig. 9:**
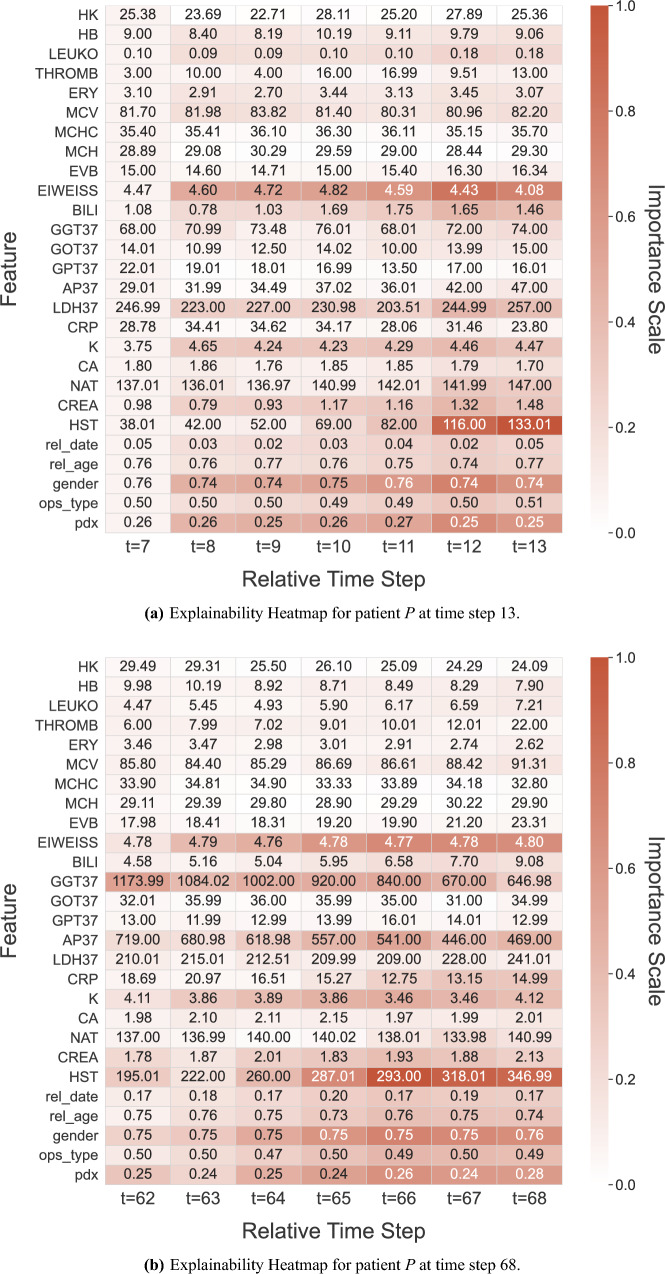
Explainability heatmaps for patient *P* at time steps (**a**) 13 and (**b**) 68, showing feature attribution scores over the last seven days before each prediction (limited to $t{-}6$ for clarity).

To investigate the factors underlying these risk elevations, we analyzed the corresponding explainability heatmaps (EXHs), shown in Fig. 9. These visualizations display feature attribution scores over time, with each row corresponding to a laboratory parameter and each column representing a time step, with color intensity indicating the magnitude of the contribution to the predicted mortality risk. For visualization purposes, the EXHs are restricted to the last seven days prior to each prediction, while the model itself operates on the full 14-day input window.

During the first risk increase at day 13, the models assigned high importance to elevated lactate dehydrogenase (LDH37) and creatinine (CREA). From a clinical perspective, this pattern was compatible with tissue damage and impaired renal function. In contrast, during the second risk escalation at day 68, these parameters became less important, while markers such as alkaline phosphatase (AP37) and gamma glutamyl transferase (GGT37) gained importance. In the post-transplant setting, such a pattern is consistent with hepatobiliary dysfunction, for example in the context of cholestasis, drug related liver injury, or liver involvement in GvHD.

These interpretations are not intended to constitute definitive diagnoses, rather than illustrating how the explanations may indicate distinct clinically relevant events associated with increases in mortality risk, even within the same patient. This further indicates that the models interpret laboratory parameters within a variable context rather than relying on a fixed feature set, thereby capturing the evolving course of post-transplant risk factors. The observed patterns were correlated with clinically recognized complications, such as organ dysfunction or inflammatory responses, suggesting that the explanations align with established medical knowledge. More generally, these results suggest that EXHs can support interpretation of model predictions in terms of clinically meaningful feature patterns and may help relate increases in predicted mortality risk to clinical reasons of patient deterioration. Establishing more systematic links between recurring explanation patterns and specific clinical scenarios remains an important direction for future work and could further improve the clinical utility of the proposed monitoring framework.

#### Providing feature importances by aggregating explainability heatmaps

**Fig. 10:**
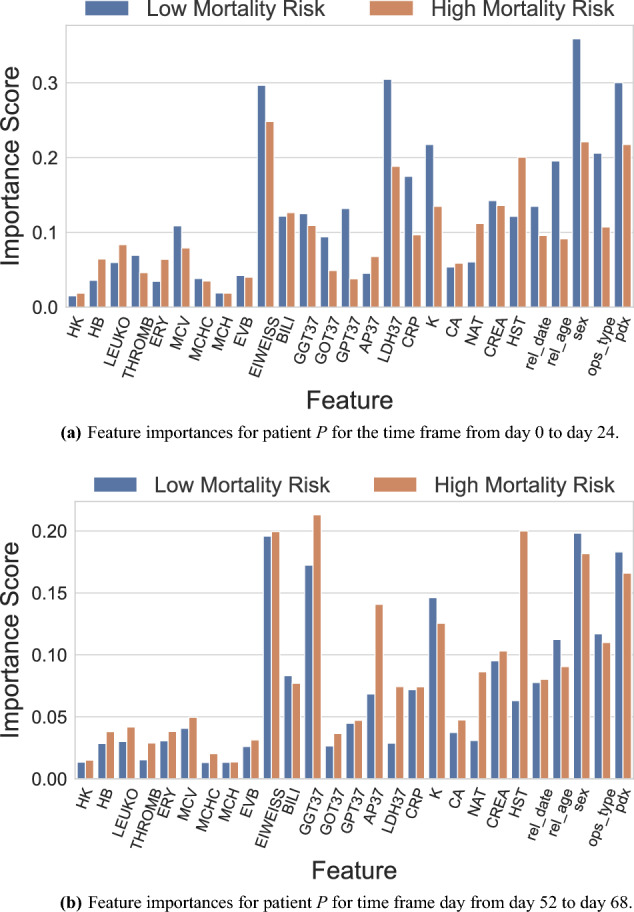
Aggregated feature importances for two time frames of patient *P*, corresponding to (**a**) the first and (**b**) the second major increases in predicted mortality risk.

In addition to subsequence-level explanations, the proposed framework enables aggregation of attribution scores across features to obtain a more general and compact representation of the laboratory parameters driving changes in predicted mortality risk. Figure 10 illustrates this approach for patient *P*. These aggregated explanations are visualized as bar plots, in which features are shown on the x-axis and bar height indicates the average attribution magnitude. Bars associated with low and high mortality risk predictions that are below and above 0.5 are distinguished by blue and orange coloring, respectively. These summaries are intended as an exploratory complement to the detailed EXHs by offering a higher-level view of model behavior. Beyond individual patient-level analyses, the same aggregation approach can also be applied across multiple patients or predefined cohorts, such as demographic subgroups or clinically defined populations, to enable identification of group-specific importance patterns (An illustrative example is provided in n Figure F1).

During the first critical phase of patient *P*, the most influential laboratory parameters comprised total protein (EIWEISS), lactate dehydrogenase (LDH37), C-reactive protein (CRP), and urea (HST), which is consistent with a pattern of inflammation, tissue damage, and impaired metabolic or renal function. In contrast, during the later phase, the models primarily attributed increased risk to urea (HST), alkaline-phosphatase (AP37), and gamma-glutamyl-transferase (GGT37), suggesting a shift toward renal and hepatobiliary involvement, while total protein (EIWEISS) remained important overall. Also, we noticed that sex (gender) and principal diagnosis (pdx) contributed noticeably to the predictions. While this could raise concerns about potential bias, we did not observe clear discrepancies between predicted and observed mortality across sex and diagnosis groups in our evaluation.

Overall, the change in aggregated feature importance between the two high-risk phases of patient *P* suggests that different laboratory parameters contributed to them. In contrast to the EXHs for individual time steps, the aggregated view can highlight which features remained relevant across a broader temporal episode of changing mortality risk.

### Assessment of model predictions and explainability by an expert physician

To assess the practical relevance and interpretability of the proposed monitoring framework, we conducted a structured case review with an expert physician who had more than three decades of clinical experience in HSCT treatment. The physician evaluated the predicted mortality risk trajectories and corresponding explanations for 20 patients, comprising true positives, false positives, false negatives, and true negatives. The reviewed cases were selected to cover both correctly identified and challenging clinical scenarios, including cases with rising risk, deteriorating clinical status, and apparent model failures.

Throughout the case study, the physician was able to understand and interpret the mortality risk progression curves and corresponding explanations without extensive guidance. Across the reviewed cases, many increases in predicted mortality risk were judged to be clinically plausible and were associated with changes in laboratory parameters that suggested evolving organ dysfunction, inflammatory deterioration, impaired hematopoietic recovery, or general worsening of the patient’s condition. In several cases, periods of increased predicted risk were followed by improvement in both laboratory profiles and the risk curves. The physician interpreted these subsequent declines in risk as being compatible with clinical stabilization and with the effects of clinical intervention.

An illustrative example is the patient shown in Fig. 7, who died outside the ICU. When reviewing the laboratory features used by the model, the physician judged the patient to have increased mortality risk, in alignment with the framework’s prediction. He noted that he would have considered ICU admission around day 81, contrary to the action that was actually taken. From a clinical perspective, he identified the later course as a rapidly worsening relapse with severe overall deterioration, which was well aligned with the model’s high-risk predictions.

A contrasting example is patient 448, who was not identified as high-risk by any model despite dying early after transplantation. For this patient, both the physician and the models assessed the laboratory values during the early course as largely unremarkable. Even when the inflammation parameter CRP increased slightly, the physician considered the finding noticeable but not indicative of high mortality risk. Based on the available laboratory measurements alone, he was unable to identify a clear high-risk state or infer a likely cause of death, suggesting that imminent deterioration may not always be apparent from the recorded laboratory features alone.

The expert also evaluated patient *P* (id: 664, Fig. 8), focusing specifically on the laboratory parameters during the two periods of increased mortality risk. He independently judged them to be clinically relevant, confirming alignment with the framework’s prediction. During the first increase, however, he would still have assigned the patient a good chance of survival, whereas the framework had already predicted a mortality risk exceeding $70\%$. During the later increase, the physician judged the laboratory profile as clearly critical and evaluated the patient as being at severe mortality risk. Thus, in both instances, the physician considered the increasing model risk clinically meaningful, while the framework appeared to indicate deterioration before the physician assessed the patient as being in a clearly critical condition.

Across the reviewed cases, the physician found the individual explainability heatmaps and aggregated feature importances to be in general understandable and medically plausible. The highlighted importance scores often corresponded to laboratory changes that were considered relevant in the clinical interpretation of the case. At the same time, differences between model and physician reasoning became apparent. The models frequently assigned high importance to features such as urea (HST) and total protein (EIWEISS), whereas the physician often focused more strongly on leukocytes (LEUKO) and thrombocytes (THROMB), particularly in cases suggestive of relapse or impaired hematopoietic recovery. In particular, total protein remained consistently important to the models but was not always directly interpretable to the physician, although he considered it a clinically meaningful surrogate of general organ related deterioration. Thus, even when the physician and the models agreed on the overall mortality risk, they did not always rely on the same laboratory parameters to arrive at this assessment.

Taken together, the expert review suggests that the proposed framework captured clinically meaningful changes in patient condition in many cases and that the corresponding explanations can support interpretation of rising risk scores. At the same time, the reviewed cases also showed clear limitations, especially when deterioration was not adequately reflected in the available laboratory features or when the clinical interpretation depends strongly on parameters that received less emphasis by the model. These findings support the clinical plausibility of the monitoring framework as a proof of concept while also highlighting the need for future studies with more detailed clinical annotations, including recorded complications and interventions.

### Validation with an external cohort from MIMIC-IV

To assess whether the observed performance generalizes beyond the UKD cohort, we evaluated the XCM models on an external cohort derived from MIMIC-IV (see Section MIMIC-IV Cohort for details). This cohort comprised 182 patients, of whom 10 died within the first 100 days post-HSCT.

At the subsequence level, the XCM models achieved an AUROC of $84\%$, a sensitivity of $22\%$, and a specificity of $99\%$. Thus, the models retained good discrimination between high-risk and low-risk periods on the external cohort, although sensitivity was lower than in the UKD cohort. At the patient level, the models achieved an AUROC of $96\%$, a sensitivity of $60\%$, and a specificity of $97\%$, i.e., six of the ten patients who died were accurately identified as high-risk before death. Among the four patients who were missed, the predicted mortality risk increased before death in two cases but did not exceed the predefined threshold for an alert, similar to what was observed in the UKD cohort. False alerts were less frequent than in the UKD cohort, indicating that high predicted risk remained relatively specific. This may in part reflect differences in cohort composition, since the MIMIC-IV cohort was derived exclusively from ICU patients.

Overall, these findings show that the proposed monitoring framework retained strong patient-level stratification performance on an independent external cohort, despite differences in cohort composition and missing information for some input features (as described in “MIMIC-IV cohort” section). Although the number of death events in the external cohort remained limited, the observed mortality frequency was comparable to that of the UKD cohort and reflected the expected class distribution for this clinical use case. Nevertheless, further external validation on larger cohorts is needed in the future to assess generalizability more comprehensively.

## Discussion and future work

This work introduces a proof-of-concept framework for continuous, explainable monitoring of short-term mortality risk during the first 100 days after allogeneic HSCT. It uses routinely collected laboratory data to generate time-resolved, patient-specific risk trajectories that can be updated with subsequent measurements throughout the course of the treatment.

The framework showed strong discrimination between survivors and decedents at both the subsequence and patient level. Further, the analyses suggest that the framework yields clinically meaningful early warnings, as patient-level risk trajectories often increased with a lead time of more than seven days before death rather than only at the terminal stage, including in patients without documented ICU stay. Although this does not show that earlier intervention would have changed outcomes, it demonstrates the potential of the framework as warning system to support decisions on escalation of clinical treatment. The continuous monitoring approach can therefore complement existing longer-term prognostic models by capturing short-term adverse changes in patient health. Additionally, the explainability component may increase the framework’s practical utility relative to static risk scores by allowing clinicians to inspect which laboratory patterns contributed to the risk estimate or to an episode of changing risk and further to relate individual patterns to other patients or populations.

The case study with the expert physician supports the clinical plausibility of the predicted risk trajectories and explanation patterns. In general, the physician was able to relate increases in predicted risk to clinically meaningful changes in laboratory parameters, and in some cases the framework reflected deterioration even before the situation was judged clearly critical by the physician. This suggests that the framework is able to complement clinician judgment and to increase awareness by making changes in mortality risk visible across time, in contrast to using laboratory assessments alone. Nonetheless, the study also revealed limitations such as the unexpected death of patient 448, which remained difficult to interpret from the limited set of laboratory parameters as well as some cases where the physician and the model agreed on overall risk but differed in which parameters appeared most relevant. Overall, the evaluation suggests that the trajectories and explanations were generally intuitive and useful for clinical interpretation.

An hypothesis that emerged from the expert evaluation and subsequent discussion is that the six most important laboratory parameters considered by the models may reflect broader biological axes of early post-transplant deterioration. In particular, bilirubin (BILI) and lactate dehydrogenase (LDH37) may indicate hepatic, endothelial, or cellular injury, creatinine (CREA) and urea (HST) may reflect renal dysfunction and systemic stress, and C-reactive protein (CRP) together with total protein (EIWEISS) may indicate inflammatory or catabolic states. This could explain the strong performance of the framework despite its compact feature set, although this interpretation will require validation in future studies that combine system outputs with systematically documented clinical events.

The external evaluation on the MIMIC-IV cohort showed that the framework retained meaningful patient-level stratification in an independent dataset despite substantial differences in clinical setting, feature availability, and documentation practice. This suggests that our monitoring framework is robust to relevant cross-cohort variation and can transfer beyond the original German single-center cohort to HSCT patients identified in a large US critical care database. Although multiple laboratory parameters required approximation during preprocessing and the external cohort does not replace dedicated multi-center validation, these results provide encouraging evidence for the robustness and transferability of the approach across centers.

Since the framework is based on routinely collected laboratory parameters, a prospective application would not require additional diagnostic procedures or documentation beyond standard monitoring practice. Moreover, the inputs and outputs remain interpretable within the clinicians’ domain of expertise and do not require detailed knowledge of the underlying architecture of the models. Therefore, the framework is best understood as an additional decision support signal that may guide decisions on closer monitoring, readmission, or escalation of care after HSCT. The explanation module should be understood as highlighting the features most relevant to the models, enabling clinicians to validate their outputs more easily.

Multiple limitations should be considered when interpreting the present results. Model development was based primarily on a single center cohort collected over a long period (2004-2019), during which transplant practice, supportive care, and structured data availability may have changed. As a result, the framework was limited to laboratory parameters and a small set of demographic features that were available in a sufficiently consistent form across patients. Other clinically relevant factors such as GvHD, conditioning details, infection status, medication exposure, comorbidity, vital signs, and cause-specific mortality labels were not included due to limitations in data availability. This may have limited detection of specific deterioration patterns, including relapse versus non-relapse mortality, and contributed to cases where the available laboratory data were not sufficient to capture increasing mortality risk. While the expert evaluation supports the clinical plausibility of the predicted trajectories and explanations, prospective studies are required to assess their value for clinical decision making in practice.

Future work should focus on prospective evaluation of the framework in clinical monitoring workflows. Such studies could assess how dynamically updated risk trajectories support physician decision-making in clinical practice, including decisions on closer monitoring, readmission, or escalation of care. They could also examine how consistently the explanations are judged clinically meaningful across a broader set of physician-reviewed cases. In addition, prospective studies would allow more detailed documentation of clinical events, interventions, complications, and causes of death, addressing important limitations of the present retrospective analysis. Another important direction is expansion to a richer feature set, which may improve performance, clinical interpretability and help capture deterioration patterns that are not fully reflected by the current features. Alternative alert strategies based on sustained or rising risk thresholds, rather than threshold crossing alone, could also be explored. Evaluation in larger and more recent cohorts may further improve assessment of robustness, calibration, and transferability under contemporary transplant practice.

## Conclusion

In this proof-of-concept study, we introduced a framework for continuous and explainable monitoring of short-term, individual, all-cause mortality risk within 100 days after allogeneic HSCT. Our framework is based on 22 routinely collected laboratory parameters and five demographic features.

Results from 891 patients from the University Hospital Düsseldorf showed that the framework provides patient-specific trajectories of short-term mortality risk that are updated daily and enable effective stratification of patients into high and low-risk groups. Further, mortality risk is often increased more than seven days before death, suggesting potential for early warning and clinical intervention. The visual design and explanations support transparency by highlighting laboratory patterns considered as important by the model and allowing predictions to be interpreted by physicians with their medical domain knowledge. The evaluation of 20 cases with an expert physician suggested that the predicted risk trajectories and highlighted laboratory patterns were clinically plausible. External evaluation on an independent MIMIC-IV cohort with 182 patients further suggested that our monitoring framework retained meaningful stratification despite differences in clinical setting and data availability.

Overall, the proposed framework outlines a promising direction for continuous monitoring and AI-based decision support after HSCT. By enabling ongoing assessment from routinely collected data, it may support closer monitoring, readmission, or escalation of care. Further prospective evaluation with richer clinical event documentation will be important to determine its value in routine practice.

## Supplementary Information

Supplementary Information.

## Data Availability

The results of our experiments and model weights generated and analysed during the current study are available in following GitHub repository: https://github.com/niruc100/explainable_mortality_risk_monitoring_hsct. Results can also be reproduced in the browser using the Code Ocean repository: https://codeocean.com/capsule/7224593/tree/v3, which additionally provides an interactive demo for running the models on previously unseen data. The raw patient data used in this study are not made publicly available for ethical and privacy reasons; access may be granted by the authors upon reasonable request and with appropriate institutional approvals. Requests for data access should be directed to Sergej Korlakov (korlakov@hhu.de).

## Code availability

Code and trained models are available on GitHub (https://github.com/niruc100/explainable_mortality_risk_monitoring_hsct), and an executable browser demo is available on Code Ocean (https://codeocean.com/capsule/7224593/tree/v3).
