# Supplementary material for: Continuous predictive mortality risk monitoring after allogeneic hematopoietic stem cell transplantation
Source: Sci Rep. 2026 Jun 18;16:19025. doi: 10.1038/s41598-026-57782-x (PMC13280382; doi:10.1038/s41598-026-57782-x)
Supplement: Supplementary file 1 — Supplementary Information. [file 41598_2026_57782_MOESM1_ESM.pdf]

Supplementary Information

Continuous Predictive Mortality Risk Monitoring After Allogeneic Hematopoietic Stem Cell Transplantation

Nick Rucks*, Sergej Korlakov*, Sebastian Alexander Scharf, Anna Rommerskirchen, Rainer Haas, Stefan Conrad

*These authors contributed equally to this work

Supplementary Data

The tables below summarize the MIMIC-IV itemids used to map laboratory features and the ICD procedure codes used to identify hematopoietic stem cell transplantation procedures in the external cohort.

Supplementary Table S1. MIMIC-IV itemids used for laboratory feature extraction.

**Feature**	**MIMIC-IV itemids**
Alkaline phosphatase at 37°C (AP37)	50863, 53086
Bilirubin (BILI)	50885, 53089
Calcium (CA)	50893
Creatinine (CREA)	52546, 50912
C-reactive protein (CRP)	50889
Total protein (EIWEISS)	50976, 53096
Erythrocytes (ERY)	51279
Erythrocyte volume distribution (EVB)	51277
Gamma-glutamyl transferase at 37°C (GGT37)	50927
Glutamic-oxaloacetic transaminase at 37°C (GOT37)	50878,53088
Glutamate pyruvate transaminase at 37°C (GPT37)	50861, 53084
Hemoglobin (HB)	51222
Hematocrit (HK)	51221
Urea (HST)	51006,52647
Potassium (K)	52610,50971
Lactate dehydrogenase at 37°C (LDH37)	50954
Leukocytes (LEUKO)	51300,51301
Mean corpuscular hemoglobin (MCH)	51248
Mean corpuscular hemoglobin concentration (MCHC)	51249
Mean corpuscular volume (MCV)	51250
Sodium (NAT)	52623, 50983
Thrombocytes / Platelets (THROMB)	51265

Supplementary Table S2. ICD procedure codes used to identify HSCT patients in MIMIC-IV.

ICD procedure code
30263Y1
30233Y1
4105
4108
30253Y1
30243Y1
30233Y2
30233Y3
30233Y4
30233G2
30233G3
30233G4
30233U2
30233U3
30233U4

Supplementary Table S3. Descriptive statistics for diagnosis categories across the UKD and MIMIC-IV cohorts. For each diagnosis category, the table reports the diagnosis behind the ICD code, a short description, and the number of recorded measurements. Diagnoses were derived from structured medical claims data and are prone to coding errors and ambiguities.

Diagnosis	Short Description	UKD Number of Measurements	MIMIC-IV Number of Measurements
Myeloid leukaemia	Malignant blood cancer arising from myeloid precursor cells, including acute and chronic myeloid leukaemia-type entities.	359	55
Myelodysplastic syndromes	Bone-marrow disorders with ineffective blood-cell production and dysplasia.	146	46
Lymphoid leukaemia	Malignant lymphoid blood cancer including acute and chronic lymphoid leukaemia-type diseases.	102	26
Monocytic leukaemia	Leukaemia involving monoblastic and monocytic cell lines.	67	0
Non-follicular lymphoma	Non-Hodgkin lymphoma category including diffuse large B-cell lymphoma, mantle-cell lymphoma, Burkitt lymphoma, and related entities.	41	6
Plasmocytoma and malignant plasma-cell neoplasms	Plasma-cell malignancies such as multiple myeloma, plasma-cell leukaemia, and plasmacytoma.	38	9
Other neoplasms of uncertain or unknown behaviour of lymphoid, haematopoietic and related tissue	Haematologic and lymphoid neoplasms of uncertain or unknown behaviour, including myeloproliferative conditions such as polycythaemia vera and myelofibrosis.	34	5
Follicular lymphoma	Follicle-centre B-cell lymphoma.	25	7
Mature T/NK-cell lymphomas	Mature T-cell or NK-cell lymphomas, including mycosis fungoides and related entities.	16	4

Diagnosis	Short Description	UKD Number of Measurements	MIMIC-IV Number of Measurements
Other aplastic anaemias	Bone marrow failure syndromes causing insufficient blood-cell production.	14	3
Other leukaemias of specified cell type	Includes specified rare leukaemia subtypes such as acute erythroid and megakaryoblastic leukaemia.	13	0
Leukaemia of unspecified cell type	Leukaemia where the specific lineage is not defined.	9	1
Hodgkin lymphoma	Malignant lymphoma of the lymphatic system with Hodgkin-type pathology.	5	2
Other specified types of T/NK-cell lymphoma	Other specified mature T-cell or NK-cell lymphoma entities.	5	0
Acquired pure red cell aplasia	Bone marrow underproduces red blood cells, causing anaemia.	3	0
Other and unspecified malignant neoplasms of lymphoid, haematopoietic and related tissue	Miscellaneous malignant blood and immune-cell tumours not otherwise classified.	2	0
Bacterial infection of unspecified site	Bacterial infection where the body site is unspecified.	2	0
Other and unspecified types of non-Hodgkin lymphoma	Other or unspecified non-Hodgkin lymphoma.	2	13
Malignant neoplasm of kidney, except renal pelvis	Kidney cancer arising in the kidney tissue rather than the renal pelvis.	1	0
Pyogenic arthritis	Bacterial joint infection causing an acutely inflamed, painful, swollen joint.	1	0
Cutaneous abscess, furuncle and carbuncle	Localized pus-forming bacterial skin and soft-tissue infection.	1	0
Other specified diseases with participation of lymphoreticular and reticulohistiocytic tissue	Includes immune and histiocytic disorders such as haemophagocytic lymphohistiocytosis.	1	0
Complications of cardiac or vascular prosthetic devices, implants and grafts	Mechanical, infectious, or inflammatory complications of cardiac or vascular implants.	1	0
Acute renal failure	Sudden decline in kidney function.	1	0
Acute tonsillitis	Acute inflammation or infection of the tonsils.	1	0
Aspergillosis	Fungal infection caused by *Aspergillus* species.	1	0
Malignant immunoproliferative diseases	Malignant disorders of immune-cell proliferation, including Waldenström macroglobulinaemia and related diseases.	0	1
Encounter for antineoplastic chemotherapy	Administrative treatment encounter code indicating chemotherapy rather than a disease diagnosis.	0	3
Complications of stem cell or bone-marrow transplant	Failure, rejection, graft-versus-host disease, infection, and related stem cell or bone-marrow transplant complications.	0	4
Other secondary thrombocytopenia	Low platelet count secondary to another disease or treatment.	0	1

**Diagnosis**	**Short Description**	**UKD Number of Measurements**	**MIMIC-IV Number of Measurements**
Zygomycosis / mucormycosis	Invasive fungal infection caused by zygomycetes or mucorales.	0	1
Poisoning by narcotics and psychodysleptics	Toxic exposure or poisoning involving opioids, hallucinogens, or related substances.	0	1
Sepsis, unspecified / other sepsis	Severe systemic infection, with unspecified organism at the category level.	0	1
Other disorders involving the immune mechanism	Miscellaneous immune-system disorders not elsewhere classified.	0	1
Agranulocytosis and neutropenia	Abnormally low neutrophil count, including agranulocytosis.	0	1

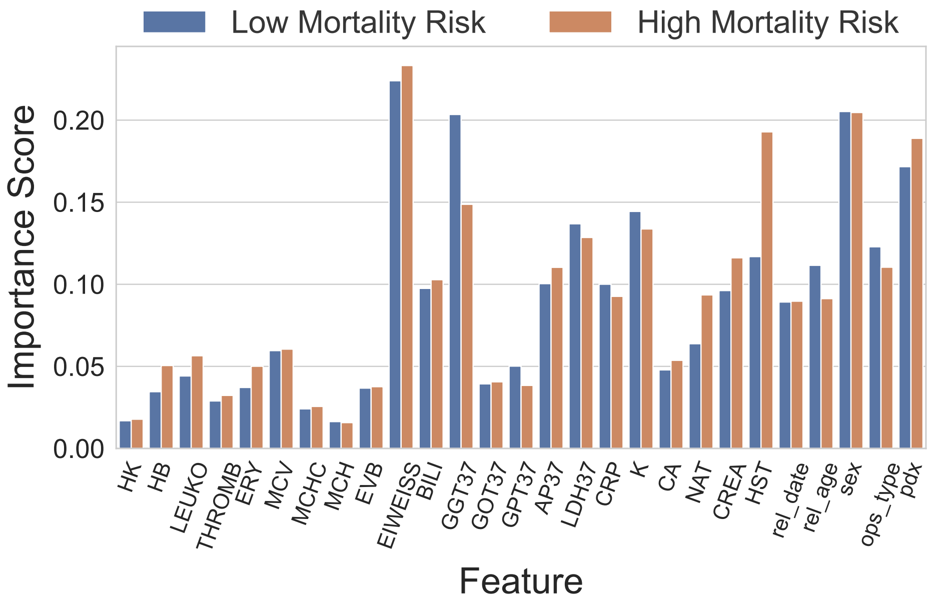

(a) Aggregated feature importance for patient P.

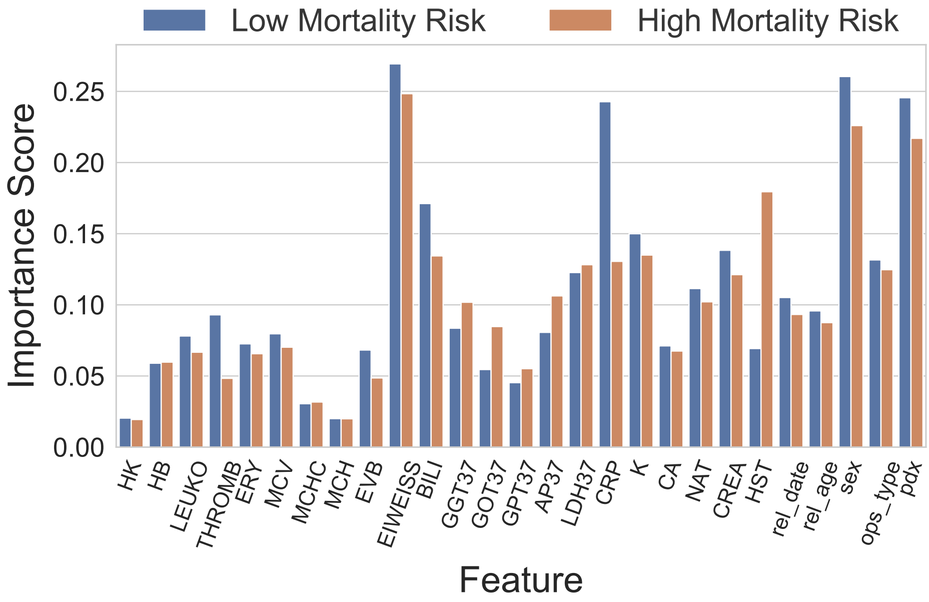

(b) Aggregated feature importance for entire patient population.

Supplementary Figure F1. Aggregated feature importances for patients and entire populations.
